# Cerebral perfusion and metabolism with mild hypercapnia vs. normocapnia in a porcine post cardiac arrest model with and without targeted temperature management

**DOI:** 10.1016/j.resplu.2024.100604

**Published:** 2024-03-12

**Authors:** Hilde Karlsen, Runar J Strand-Amundsen, Christiane Skåre, Morten Eriksen, Vidar M Skulberg, Kjetil Sunde, Tor Inge Tønnessen, Theresa M Olasveengen

**Affiliations:** a Department of Research and Development and Institute for Experimental Medical Research, Oslo University Hospital, Norway; b Institute of Clinical Medicine, University of Oslo, Oslo, Norway; c Department of Clinical and Biomedical Engineering, Oslo University Hospital, Oslo, Norway; d Department of Anesthesia and Intensive Care Medicine, Oslo University Hospital, Oslo, Norway; e University of Oslo, Oslo, Norway; f Institute for Experimental Medical Research, Oslo University Hospital, Oslo, Norway

**Keywords:** Resuscitation, post-ROSC, Cardiac arrest, Ventilation, Hypercapnia, Experimental study, Target temperature management, TTM, Hypothermia, Microdialysis, Cerebral perfusion pressure, Haemodynamic, Post-cardiac arrest care

## Abstract

**Aim:**

To determine whether targeting mild hypercapnia (PaCO_2_ 7 kPa) would yield improved cerebral blood flow and metabolism compared to normocapnia (PaCO_2_ 5 kPa) with and without targeted temperature management to 33 °C (TTM33) in a porcine post-cardiac arrest model.

**Methods:**

39 pigs were resuscitated after 10 minutes of cardiac arrest using cardiopulmonary bypass and randomised to TTM33 or no-TTM, and hypercapnia or normocapnia. TTM33 was managed with intravasal cooling. Animals were stabilized for 30 minutes followed by a two-hour intervention period. Hemodynamic parameters were measured continuously, and neuromonitoring included intracranial pressure (ICP), pressure reactivity index, cerebral blood flow, brain-tissue pCO_2_ and microdialysis. Measurements are reported as proportion of baseline, and areas under the curve during the 120 min intervention period were compared.

**Results:**

Hypercapnia increased cerebral flow in both TTM33 and no-TTM groups, but also increased ICP (199% vs. 183% of baseline, *p* = 0.018) and reduced cerebral perfusion pressure (70% vs. 84% of baseline, *p* < 0.001) in no-TTM animals. Cerebral lactate (196% vs. 297% of baseline, *p* < 0.001), pyruvate (118% vs. 152% of baseline, *p* < 0.001), glycerol and lactate/pyruvate ratios were lower with hypercapnia in the TTM33 group, but only pyruvate (133% vs. 150% of baseline, *p* = 0.002) was lower with hypercapnia among no-TTM animals.

**Conclusion:**

In this porcine post-arrest model, hypercapnia led to increased cerebral flow both with and without hypothermia, but also increased ICP and reduced cerebral perfusion pressure in no-TTM animals. The effects of hypercapnia were different with and without TTM.

(Institutional protocol number: FOTS, id 14931)

## Introduction

Sudden, unexpected out-of-hospital cardiac arrest (OHCA) is a leading cause of death affecting approximately 275 000 Europeans every year.^1^, ^2^ Successful initial resuscitation is followed by a complex pathophysiological process initiated by regional and global ischemia and reperfusion termed the post-cardiac arrest syndrome (PCAS).^3^ This syndrome may cause persistent low cerebral perfusion,^4^, ^5^, ^6^, ^7^, ^8^ leaving the brain vulnerable to injury.^9^ As two-thirds of all hospital deaths after cardiac arrest are associated with hypoxic-ischemic brain injury,^10^, ^11^, ^12^ there is an urgent need for new therapeutic options to prevent secondary brain injury.^13^

Mild therapeutic hypercapnia has been suggested as a potential strategy to reduce brain injury by increasing cerebral blood flow after cardiac arrest.^14^, ^15^Hypercapnia has also been shown to have anti-convulsive, anti-inflammatory and anti-oxidant properties in experimental studies.^16^, ^17^, ^18^ Despite these initial promising findings, the recently published TAME trial (Targeted Therapeutic Mild Hypercapnia After Resuscitated Cardiac Arrest) failed to demonstrate any benefit from 24 hours of hypercapnia on favourable neurological outcomes.^19^ In this study, we propose to explore the effects of hypercapnia on cerebral circulation and metabolism after cardiac arrest in our experimental cardiac arrest model. Gaining insight into how hypercapnia affects the pathophysiology post-arrest might shed some light on the neutral clinical trial and enable development of more tailored approaches to manage ventilation and circulation in post-cardiac arrest patients.

Animal models provide important insights into therapeutic interventions by offering standardisation between groups, and invasive monitoring that surpasses anything possible in the clinical setting. In this experimental porcine study, we aimed to explore potential differences in brain perfusion, metabolism, and autoregulation in animals where PaCO_2_ was targeted to 7 vs. 5 kPa after 10 minutes of untreated cardiac arrest, both with and without targeted temperature management (TTM) to 33 °C.

## Methods

### Study design

This was a non-blinded randomized controlled trial (RCT) in pigs performed at the Institute for Experimental Medical Research, Oslo University Hospital, Ullevål, Norway, consisting of two experimental series. Animals with return of spontaneous circulation (ROSC) were included and randomised to mild hypercapnia (PaCO_2_ 6.5–7.0 kPa) or normocapnia (PaCO_2_ 5.0–5.5 kPa) with TTM to 33 °C (TTM33) or no-TTM.

Norwegian National Animal Research Authority approved the study, and all the involved staff were certified with Federation of Laboratory Animal Sciences Associations category C.^20^ The study was performed in compliance with EU Directive 2010/63/EU for animal experiments, with reporting in accordance with ARRIVE guidelines.^21^

### Animal preparation, instrumentation and monitoring

We used crossbreed Norwegian Landrace pigs of either sex (approximately 35 kg), kept overnight with free access to water and food. The animals were pre-medicated, anaesthetized and surgically prepared, using the same equipment (Table S1 supplemental material) as previously described.^22^

In brief, it included tracheostomy, urine catheter with thermometer via cystotomy. Left and right intraventricular pressure catheters were placed through the left carotid artery and right internal jugular vein respectively. An ultrasound flowmeter probe was placed around the carotid artery and a pulmonary artery catheter was placed through the right femoral vein. A fluid filled polyethylene catheter was inserted into the lower abdominal aorta from the right femoral artery. Both catheters were used for continuous pressure monitoring and blood gas monitoring. Cardiopulmonary bypass (CPB) cannulas were located through the right external jugular vein and left femoral artery and controlled by x-ray. In TTM33 animals, a cooling catheter was inserted in the left femoral vein for invasive management, connected to the CPB.

We made cranial burr hole for placement of Doppler flowmetry probe (for measurement of cerebral blood flow (CBF)), pressure transducer catheter (for monitoring of intracranial pressure (ICP)), microdialysis catheters (for analysis of lactate [mM], pyruvate [µM], lactate/pyruvate-ratio, glutamate [µM], glucose [mM], and glycerol [µM]), and IscAlert pCO_2_ sensors (to detect changes in tissue pCO_2_ (PtCO_2_) from baseline). CO_2_ is produced in the brain by metabolism in the Krebs cyclus. With decreasing blood flow PtCO_2_ will increase under aerobic conditions. Under anaerobic conditions lactic acid is buffered by bicarbonate to carbonic acid and PtCO2 increased rapidly 3 – 10-fold and is a reliable marker of ischemia.^23^, ^24^, ^25^, ^26^, ^27^, ^28^

The animals were mechanically ventilated, and they received positive end-expiratory pressure of 5 cm H_2_O. Fraction of inspired oxygen and minute ventilation were adjusted to target SpO_2_ 95–100% and end-tidal carbon dioxide (EtCO_2_) 4.5–5.5 kPa at baseline (tidal volumes 8–12 ml/kg, respiratory rate 15–20/min and 25–30% O_2_). Defibrillation pads were placed on the thorax, connected to a defibrillator, and used for electrocardiogram monitoring.

### Experimental protocol

Following the surgical preparation and the pigs being placed on their left side, there was a 30-minutes stabilisation period, before baseline blood gas analysis and registration of all parameters were done. The pigs were connected to a Ringer’s acetate primed CPB circuit and circuitry set to stand by with clamped vascular connections. They were anticoagulated with Heparin 500 IU/kg IV to prevent blood clotting in the circuit. Ventricular fibrillation (VF) was induced by trans-thoracic current (90 V AC for 3 s), and cardiac arrest confirmed by electrocardiogram and abrupt drop in blood pressure. VF was left untreated for 10 minutes without ventilation and propofol infusion was paused. Resuscitation using CPB was initiated with a flow of 100 ml/kg/min and propofol infusion and mechanical ventilation were restarted. TTM33 was initiated by lowering the temperature of the CPB heat exchanger to 20 °C, and initiating cooling through the endovascular cooling catheter.

**Fig. 1:**
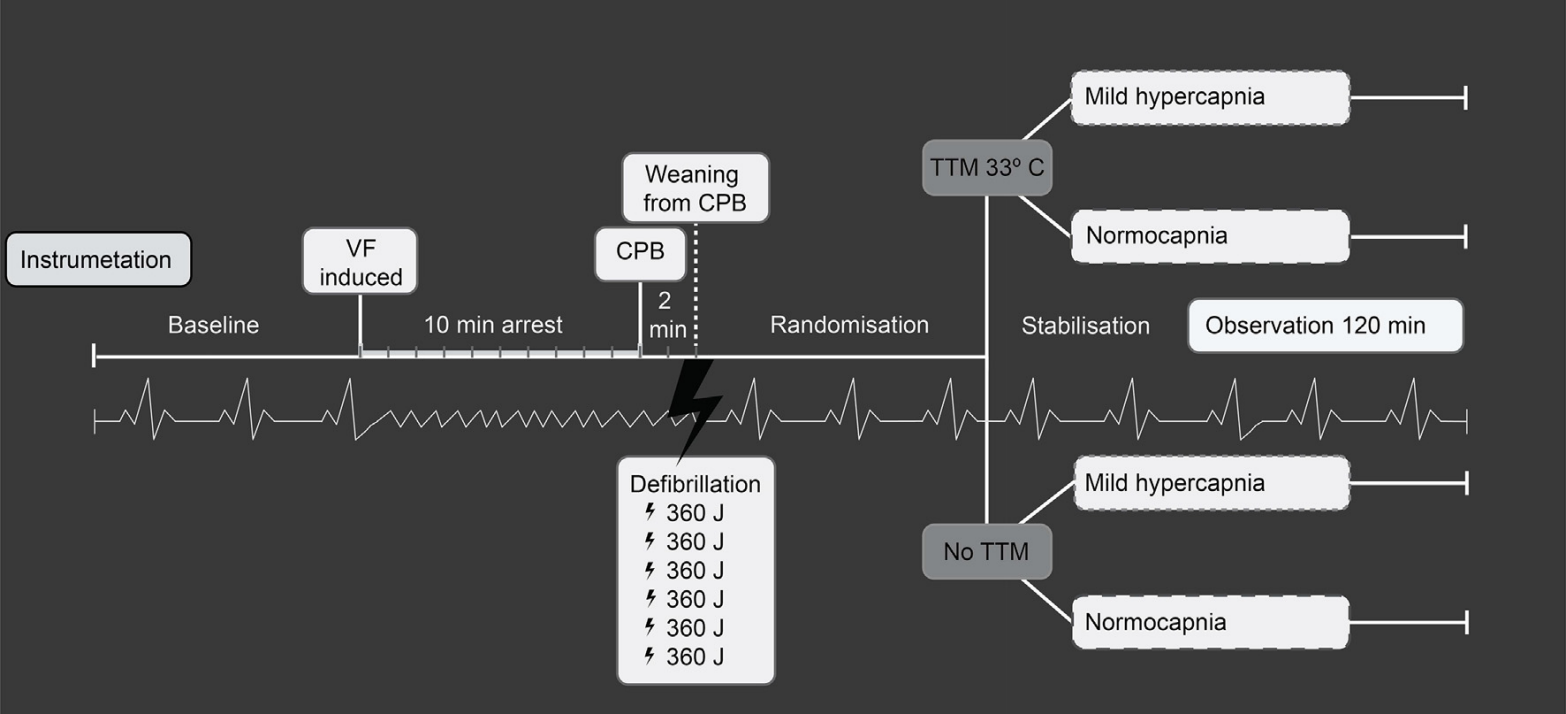
**Overview of the experiment.** Animals were anaesthetised and surgically prepared preceding to electrical induction of ventricular fibrillation (VF). VF was left untreated for 10 minutes. Resuscitation with cardiopulmonary bypass (CPB) was initiated with a flow of 100 ml/kg/min for 2 minutes before defibrillation with 360 J. Maximum 6 shock were allowed to achieve return of spontaneous circulation (ROSC). With ROSC, they were weaned off CPB by 0.5 l/min, and randomised to normocapnia or hypercapnia. The study consisted of two series, no temperature target management (No TTM), and TTM 33 °C.

After two minutes of resuscitation, defibrillation was performed with 360 Joule (J). The study protocol allowed for defibrillations every two minutes until ROSC, with a maximum of six shocks. Successful defibrillation was followed by CPB weaning with 0.5 l/min and residual blood in the circuit returned. Thereafter, the pigs were randomised to hypercapnia or normocapnia. The intervention started immediately after ROSC where a 30 min stabilization and titration period were followed by 120 minutes of observation and registration of outcome parameters. Minute ventilation was adjusted to achieve randomized PaCO_2_ target. Finally, all animals were euthanized with an intravenous injection of 50 mmol potassium chloride. An illustration of the study protocol may be found in Fig. 1.

*Measurement parameters*.

We used real time data acquisition hardware Powerlab 16/35 with Octal Bridge Amplifier (FE228) from ADInstruments o sample haemodynamic variables throughout the experiment. Cerebral perfusion pressure (CePP) was calculated as the difference between aortic and intracerebral pressure, and coronary perfusion pressure (CPP) as the difference between aortic and right atrial pressure. Data were recorded continuously and are presented with 10- and −15-minutes intervals. Pressure reactivity index (PRx), a correlation coefficient between ICP and mean arterial pressure (MAP), was calculated as the moving correlation coefficient between consecutive 5-second averages of ICP and MAP and has been used as a measure of autoregulation.^29^, ^30^

*Experimental outcomes*.

Cardiac parameters; heart rate [beats/min], MAP [mmHg], pulmonary artery pressure (PAP) [mmHg], end-diastolic left ventricular pressure (EDLVP) [mmHg], CPP [mmHg] and CO [litres/min].

Cerebral parameters; ICP [mmHg], CePP, carotid flow (CaF) [ml/min] PtCO_2_ [kPa]. CBF [arbitrary unites – AU], PRx, brain tissue microdialysis (lactate, pyruvate, LP ratio, glucose, glutamate, glycerol). CaF and CBF were considered important to assess whether increased perfusion pressure would lead to increased flow, whereas brain PtCO_2_ and microdialysis were selected to add information about brain metabolism, ischaemia and inflammation. As post arrest neurological assessments were not possible due to the study design, the reactivity indexes were selected to provide indirect information about brain function.

Relative to baseline values were chosen to reduce the effect of variation between the individual animals. Relative to baseline = ((measurement – baseline value)/baseline value).

*Determination of sample size*.

This was an explorative animal study based on a previous pig model.^22^ No formal power analysis was performed, but we expected to need between 7–12 animals per group.

### Statistical analysis

Baseline variables are reported as mean values with 95% confidence intervals where normocapnia and hypercapnia groups are compared with or without TTM33. The variables did not behave in a linear fashion with time, precluding linear regression analysis. Areas under the curves (AUC) for hypercapnia and normocapnia groups during the 120 minute intervention period were calculated for hemodynamic and metabolism parameters and compared by t-test. Actual values were used for temperature and arterial pCO_2_ to demonstrate adherence to planned interventions, but values relative to baseline were used for all other variables. Values relative to baseline were chosen because of the variability of the animals gender, size and physiology at baseline. SPSS version 26 and GraphPad Prism 9.3.1 were used for all statistical analyses. A *p*-value of less than 0.05 was considered statistically significant.

## Results

**Fig. 2:**
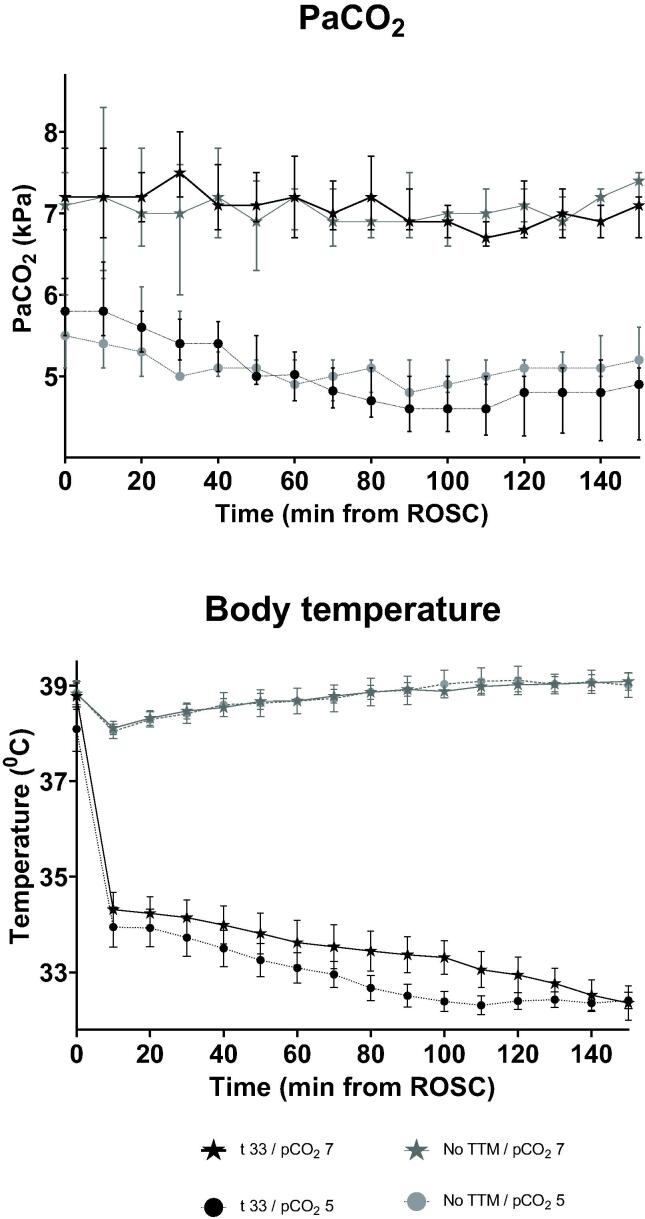
**PaCO_2_ and body temperature from return of spontaneous circulation.** PaCO_2_ levels are reported in kPa and temperature in °C. Graphs are presented as means with standard error. Areas under the curves (AUC) for hypercapnia and normocapnia groups were compared by t-test.

43 animals were needed to include 39 animals (4 pigs did not achieve ROSC), 19 randomized to hypercapnia and 20 to normocapnia. Baseline characteristics are shown in Table 1. Both temperature and PaCO_2_ targets were met prior to the end of the 30 min stabilization period, and there was good separation between the groups (Fig. 2).

**Table 1:** Baseline characteristics.

	**No TTM**		**TTM 33 °C**	
	**Normocapnia** **(*n* = 9)**	**Mild hypercapnia** **(*n* = 10)**	**Normocapnia** **(*n* = 9)**	**Mild hypercapnia** **(*n* = 11)**
Weight	35 (34, 36)	34 (33, 40)	34 (33, 35)	35 (34, 36)

**Pressure measurements**				
Heart Rate (beats/min)	96 (78, 126)	96 (80, 122)	90 (77, 137)	78 (74, 117)
Mean arterial pressure (mmHg)	103 (81, 124)	108 (92, 130)	102 (97, 114)	94 (85, 105)
Pulmonary Artery Pressure (mmHg)	14 (6, 17)	17 (14, 22)	18 (13, 23)	16 (14, 20)
End-diastolic Left ventricular Pressure (mmHg)	23 (16, 25)	20 (17, 24)	11 (7, 15)	14 (10, 19)
Intracranial Pressure (mmHg)	11 (6, 24)	8 (7, 12)	12 (6, 21)	11 (6, 18)
Cerebral Perfusion Pressure (mmHg)	89 (59, 106)	94 (82, 118)	95 (79, 105)	75 (71, 95)
Coronary Perfusion Pressure (mmHg)	65 (24, 79)	69 (52, 86)	80 (64, 88)	60 (54, 73)

**Flow and reactivity measurements**				
Cardiac Output (litres/min)	4.3 (3.8, 5.4)	4.8 (4.4, 5.4)	4.1 (3.3, 5.0)	3.4 (3.2, 4.3)
Carotid Flow (ml/min)	226 (113, 288)	271 (228, 330)	197 (150, 277)	237 (205, 309)
Pressure Reactivity Index (PRx)	0.01 (0.01, 0.14)	−0.07 (0.02, 0.19)	0.02 (-0.4, 0.28)	−0.03 (0.02, 0.21)

**Metabolism**				
SvO_2_ (%)	52 (42, 76)	60 (49, 72)	60 (51, 64)	55 (52, 61)
Brain tissue PtCO_2_ (kPa)	6.4 (5.1, 8.3)	8.2 (6.6, 9.2)	7.1 (6.4, 7.5)	6.6 (6.3, 7.6)
Brain lactate (mM)	0.8 (0.7,0.9)	0.8 (0.5, 1.1)	0.9 (0.8, 1.2)	1.2 (0.9, 1.3)
Brain pyruvate (µM)	32 (25, 51)	40 (35, 68)	55 (40, 77)	50 (35, 61)
Brain glucose (mM)	1.1 (0.9, 1.5)	1.3 (1.0, 1.8)	1.4 (1.1, 1.6)	1.1 (0.9, 1.3)
Brain glutamate (µM)	22 (17, 46)	19 (9, 29)	35 (26, 47)	32 (20, 52)
Brain glycerol (µM)	56 (33, 81)	50 (47, 64)	69 (51, 75)	61 (50, 75)
Brain Lactate/Pyruvate Ratio	24 (16, 30)	20 (12, 36)	26 (-17, 118)	22 (18, 36)
Brain temperature (°C)	38.9 (35.8, 41.4)	39.6 (38.2, 41.5)	39.2 (37.6, 40.5)	38.8 (38.7, 39.7)
Core body temperature (°C)	38.8 (38.4, 39.7)	38.9 (39.0, 39.7)	38.1 (37.1, 40.5)	38.6 (37.7, 39.3)
Blood lactate (mM/l)	0.6 (0.4, 0.7)	0.7 (0.6, 1.1)	0.9 (0.7, 1.2)	0.7 (0.6, 1.0)
Blood glucose (mM/l	4.7 (4.0, 5.8)	5.4 (4.2, 6.0)	4.9 (4.6, 5.9)	4.6 (3.8, 5.4)
EtCO_2_	5.2 (4.4, 5.8)	5.3 (4.9, 5.8)	5.2 (4.6, 5.6)	4.7 (4.7, 5.4)
Minute Ventilation (MV)	9.0 (7.7, 9.9)	11.3 (8.6, 18.2)	8.5 (8.0, 10.0)	8.5 (8.2, 9.8)
Respiration rate (per min)	20 (18, 22)	22 (20, 26)	20 (19, 22)	20 (19,22)
Arterial pCO2 (kPa)	5.3 (5.1, 5.3)	5.4 (5.0, 5.6)	5.4 (5.1, 5.5)	5.2 (4.9, 5.4)

All variables are reported as mean values with 95% confidence intervals. Pressure reactivity index as the Pearson’s correlation coefficient between intracranial and blood pressure. SvO_2_ = Mixed venous oxygen saturation. EtCO_2_ = End-tidal CO_2_.

### Pressure measurements

**Fig. 3:**
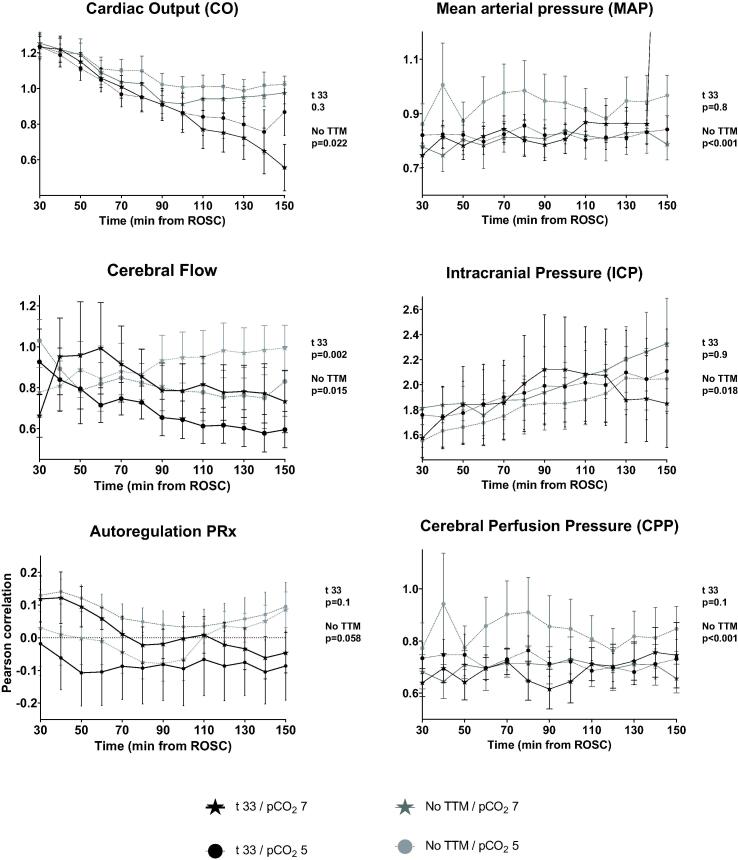
**Hemodynamic parameters from return of spontaneous circulation.** All values are reported as relative to baseline. Graphs are presented as means with standard error. Areas under the curves (AUC) for hypercapnia and normocapnia groups were compared by t-test.

In TTM33 animals there were no differences in MAP, ICP and CePP in the hypercapnia vs. normocapnia groups. In animals with noTTM the hypercapnia group had lower MAP (average 81% vs. 94% of baseline during intervention, *p* < 0.001), higher ICP (average 199% vs. 183% of baseline during intervention, *p* = 0.018) and thereby lower CPP (average 70% vs. 84% of baseline during intervention, *p* < 0.001). PRx was within the normal range for all four groups‘ indicating autoregulation was preserved during the intervention period (Table 2, Fig. 3).

**Table 2:** Dose relative to baseline during 120-minute intervention period post-ROSC.

	**No TTM**			**TTM 33 °C**		
	**Normocapnia** **(*n* = 9)**	**Mild hypercapnia** **(*n* = 11)**	***p*-value**	**Normocapnia** **(*n* = 9)**	**Mild hypercapnia** **(*n* = 10)**	***p*-value**
**Interventions**						
Arterial pCO_2_ (kPa)	5.2 ± 0.1	7.0 ± 0.1	<0.001	5.0 ± 0.1	7.1 ± 0.1	<0.001
Core body temperature(°C)	38.8 ± 0.1	38.8 ± 0.1	0.09	33.1 ± 0.2	33.6 ± 0.2	<0.001

**Pressure**						
Mean arterial pressure	0.94 ± 0.06	0.81 ± 0.05	<0.001	0.82 ± 0.03	0.83 ± 0.04	0.8
Intracranial Pressure	1.83 ± 0.13	1.99 ± 0.13	0.018	1.94 ± 0.19	1.93 ± 0.21	0.9
Cerebral Perfusion Pressure	0.84 ± 0.0.07	0.70 ± 0.05	<0.001	0.72 ± 0.04	0.69 ± 0.05	0.1
Pressure Reactivity Index (PRx)	0.07 ± 0.03	0.04 ± 0.04	0.058	0.08 ± 0.07	0.04 ± 0.03	0.1

**Flow**						
Cardiac Output	0.99 ± 0.04	0.95 ± 0.04	0.022	0.87 ± 0.06	0.84 ± 0.05	0.3
Carotid Flow	0.97 ± 0.03	0.96 ± 0.03	0.5	1.02 ± 0.03	1.02 ± 0.03	0.7
Cerebral Flow (CF)	0.81 ± 0.06	0.91 ± 0.07	0.015	0.69 ± 0.07	0.84 ± 0.09	0.002
Brain temperature	0.98 ± 0.01	1.00 ± 0.00	<0.001	0.88 ± 0.01	0.89 ± 0.00	0.005

**Metabolism**						
Brain tissue PtCO_2_	1.11 ± 0.05	1.32 ± 0.04	<0.001	0.94 ± 0.06	1.26 ± 0.03	<0.001
Brain lactate	2.39 ± 0.30	2.23 ± 0.26	0.3	2.97 ± 0.38	1.96 ± 0.23	<0.001
Brain pyruvate	1.50 ± 0.11	1.33 ± 0.07	0.002	1.52 ± 0.16	1.18 ± 0.13	<0.001
Brain glucose	1.17 ± 0.07	1.06 ± 0.09	0.011	1.06 ± 0.11	0.91 ± 0.010	0.007
Brain glutamate	0.69 ± 0.19	1.08 ± 0.46	0.026	0.77 ± 0.20	0.84 ± 0.20	0.5
Brain glycerol	3.17 ± 0.27	3.14 ± 0.30	0.9	2.86 ± 0.25	2.49 ± 0.19	0.002
Brain Lactate/Pyruvate Ratio	1.57 ± 0.16	1.62 ± 0.20	0.6	2.37 ± 0.54	1.85 ± 0.23	0.012

All variables are reported as relative to baseline except the interventions arterial pCO_2_, core temperature and pressure reactivity index. Arterial pCO_2_ is reported as kPa, core temperature as degrees Celsius, and pressure reactivity index as the Pearson’s correlation coefficient between intracranial and blood pressure. All other values are reported as mean proportions of baseline with standard errors (SEM) and calculated from the Areas Under the Curves. Areas under the curves (AUC) for hypercapnia and normocapnia groups were compared by t-test.

### Flow measurements

In TTM33 animals there were no differences in CO or CaF between groups, but CBF (average 84% vs. 69% of baseline during intervention, *p* = 0.002) and brain temperature (average 89% vs. 88% of baseline during intervention, *p* = 0.005) were increased the hypercapnia group compared to normocapnia group. In animals with noTTM the hypercapnia group had higher CBF (average 91% vs. 81% of baseline during intervention, *p* = 0.015) and brain temperature (average 100% vs. 98% of baseline during intervention, *p* < 0.001), but lower CO (average 95% vs. 99% of baseline during intervention, *p* = 0.022) compared to the normocapnia group (Table 2, Fig. 3).

### Metabolism

**Fig. 4:**
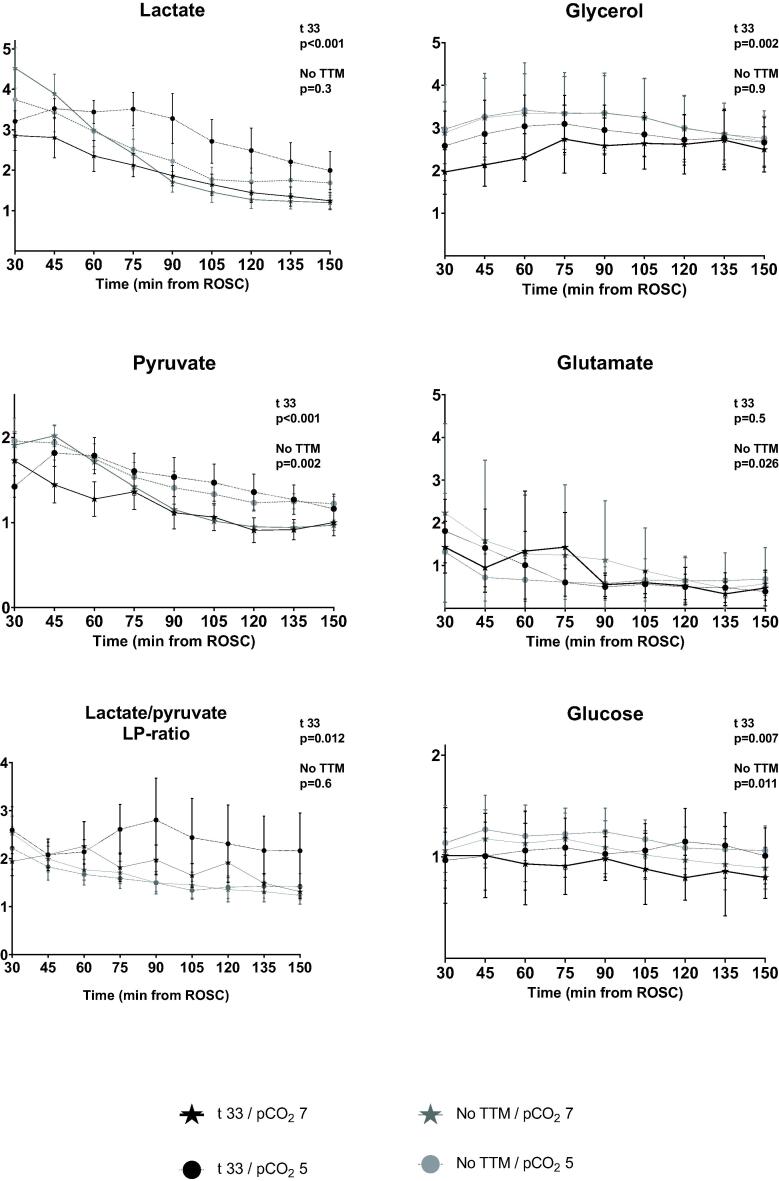
**Cerebral microdialysis from return of spontaneous circulation.** All values are reported as relative to baseline. Graphs are presented as means with standard error. Areas under the curves (AUC) for hypercapnia and normocapnia groups were compared by t-test.

In TTM33 animals, the hypercapnia group had lower lactate (average 196% vs. 297% of baseline during intervention, *p* < 0.001), pyruvate (average 118% vs. 152% of baseline during intervention, *p* < 0.001), glucose (average 91% vs. 106% of baseline during intervention, *p* = 0.007), glycerol (average 249% vs. 286% of baseline during intervention, *p* = 0.002) and LP ratio levels (average 185% vs. 237% of baseline during intervention, *p* = 0.012) compared to the normocapnia group. In the animals with noTTM, the hypercapnia group had lower pyruvate (average 133% vs. 150% of baseline during intervention, *p* = 0.002) and glucose (average 106% vs. 117% of baseline during intervention, *p* = 0.011) levels, but higher glutamate (average 108% vs. 69% of baseline during intervention, *p* = 0.026) levels compared to the normocapnia group. There was no difference in lactate (average 223% vs. 239% of baseline during intervention, *p* = 0.3). There were no significant differences in LP ratio (Table 2, Fig. 4).

## Discussion

The findings of this animal experiment provide valuable insights into the effects of hypercapnia on post-cardiac arrest physiology and suggest these effects may vary at different temperatures. While CBF and brain temperatures were higher with hypercapnia in both temperature groups as hypothesized, the effects on the systemic circulation and metabolism were temperature dependant. Lower brain tissue levels of lactate and pyruvate are indicators of less ischemia and suggests hypercapnia could be neuroprotective. These effects on lactate and pyruvate were more pronounced during TTM33 compared to noTTM in our porcine model.

Babini and colleagues performed a similar animal experiment exploring hypercapnia in a porcine post-arrest model.^31^ Their animals were allowed to survive for up to 96 hours and their main outcomes focused on neurological assessments and biomarkers of brain injury. While they found some indications of less brain injury, neurological recovery was equivalent between the two groups. As this was a survival experiment, it did not allow for extensive invasive monitoring of brain physiology and the authors unable to determine whether their intervention had improved CPP, CBF or metabolism.

Several clinical studies have observed cerebral vasoconstriction and hypoperfusion after cardiac arrest,^32^, ^33^, ^34^, ^35^ suggesting cerebral vasodilation might be an appealing therapeutic target. Clinical studies have illustrated the close relationship between cerebral vasodilatation and increased PaCO_2_ using transcranial doppler and near-infrared spectroscopy (NIRS) to show increases in middle cerebral artery velocities and cerebral oximetry with higher PaCO_2_ levels.^36^, ^37^, ^38^ Similarly, observations from laboratory studies using invasive procedures like laser doppler flow measured directly over the dura^39^ and measuring changes in the pial arteriolar diameter by a video micrometer^40^ support hypercapnia as a method to increase CBF. While increasing CBF after brain injury has appeal, it would only be useful in preventing secondary injuries if there is an energy imbalance where the brains oxygen supply does not meet the demand. A disproportionate focus on solely optimising oxygen delivery without consideration of abnormalities in oxygen diffusion or utilisation has been suggested as a possible explanation for the failure of recent trials attempts to reduce brain injury after cardiac arrest.^41^

The disappointing results of the TAME trial on neurological intact survival has many possible explanations.^19^ One of the explanations suggested by the authors of the TAME trial was that hypercapnia may have worsened cerebral oedema and elevated intracranial pressure. The TAME trial did not measure intracranial pressure, but suggested intracranial hypertension was unlikely to be common in this patient population as there was only one report of intervention-related cerebral oedema in the mild hypercapnia group. Without systematically assessing for intracranial hypertension in the clinical trial, our experimental data could suggest this might still be a risk in particular for the patients not treated with TTM33. Patients included in the TAME trial were also treated at different temperature levels (33, 36 and <37.8 °C) according to co-enrolment in the TTM2 trial^42^ and varying local protocols, and our experimental data suggests this may also have influenced the effects of hypercapnia.

Microdialysis enables the biochemical variables of the extracellular interstitial space to be monitored and provides data on substrate supply and metabolism at the cellular level in the brain.^43^ In clinical conditions, the cerebral cytoplasmic redox state is conventionally evaluated from the LP ratio obtained from intracerebral microdialysis. Compromised energy metabolism will cause a shift in the cytoplasmic redox state that is rapidly reflected in an increase of the LP ratio. In hypoxic conditions pyruvate is metabolized to lactate, and therefore high LP ratio is considered as robust indicator of anaerobic metabolism.^44^, ^45^, ^46^, ^47^ The increase in LP ratios were smaller than we had expected and could indicate that our model would benefit from a larger insult to the brain.

Although findings from animal experiments are not directly applicable to humans, they offer insights into potential explanations for underlying mechanisms driving clinical observations. These animal data pose some important questions for further clinical investigation. Specifically, could hypercapnia be beneficial in subpopulations with reduced cerebral blood flow without intracranial hypertension? Are there important and potentially complex interactions with temperature? Systemic effects of hypercapnia in normothermia included vasodilatation that led to lower MAP, cerebral perfusion pressure and cardiac output while still augmenting cerebral blood flow. TTM33 is a potent systemic vasoconstrictor that largely negated the systemic but not the cerebral vasodilatory effects of hypercapnia.

## Limitations

There are several limitations worth mentioning. Firstly, the pigs were young and otherwise healthy prior to our experiments whereas human cardiac arrest patients are often older and frequently have co-morbidities. Pigs also have a normal temperature of 39–40 °C which complicates interpretation of animal experiments cooling pigs to human targets. Additionally, cardiopulmonary bypass resuscitation is different from conventional CPR and will not precisely mirror forward blood flow generated by chest compressions. And while providing consistent and standardised blood flow, the extracorporeal circulation may contribute to the severity of the sepsis-like syndrome commonly observed after cardiac arrest.^48^ Our method for cranial burr hole did not involve an exact closed cranial window, however all catheters and invasive monitors were placed using as small a burr hole as possible to minimize leakage. We used a 30 min stabilization or calibration period prior to recording baseline – this could have been short in terms of washing out trauma from inserting microdialysis catheters. We used pigs of both sex in this study, and we did not take into consideration the differences there might be between the sexes. Lastly, few animals met criteria for autoregulatory dysfunction with PRx values below 0.2. This could indicate our model did not yield sufficient brain damage to assess effects of hypercapnia on autoregulation.

## Conclusions

In this porcine post-arrest model, hypercapnia led to increased cerebral flow both with and without TTM33, but also reduced cerebral perfusion pressure in no-TTM animals. The effects of hypercapnia were different with and without TTM.

**Institutions where the work was performed:** Institute for Experimental Medical Research, Oslo University Hospital, Oslo, Norway.

## CRediT authorship contribution statement

**Hilde Karlsen:** Writing – original draft, Visualization, Methodology, Investigation, Formal analysis, Data curation, Conceptualization. **Runar J Strand-Amundsen:** Writing – review & editing, Validation, Software, Project administration, Methodology, Investigation, Formal analysis, Data curation. **Christiane Skåre:** Writing – review & editing, Methodology, Investigation, Formal analysis, Data curation, Conceptualization. **Morten Eriksen:** Writing – review & editing, Project administration, Methodology, Investigation, Funding acquisition, Data curation. **Vidar M Skulberg:** Writing – review & editing, Software, Methodology, Formal analysis, Data curation. **Kjetil Sunde:** Writing – original draft, Supervision, Resources, Project administration, Conceptualization. **Tor Inge Tønnessen:** Writing – review & editing, Supervision, Resources, Funding acquisition, Data curation, Conceptualization. **Theresa M Olasveengen:** Writing – review & editing, Writing – original draft, Validation, Supervision, Resources, Project administration, Methodology, Investigation, Funding acquisition, Formal analysis, Data curation, Conceptualization.

## Declaration of competing interest

The authors declare the following financial interests/personal relationships which may be considered as potential competing interests: H. Karlsen: None C. Skåre: Research grant from Zoll Foundation, receieved May 2019. R.J Strand-Amundsen: Employed by Sensocure AS. M. Eriksen: None. V.M Skulberg: None. K. Sunde: None. T.I. Tønnessen: Shareholder, board member and medical advisor for Sensocure AS. T.M. Olasveengen: Board member for the Laerdal Foundation.
