# Supplementary material for: Cerebral perfusion and metabolism with mild hypercapnia vs. normocapnia in a porcine post cardiac arrest model with and without targeted temperature management
Source: Resusc Plus. 2024 Mar 12;18:100604. doi: 10.1016/j.resplu.2024.100604 (PMC10950799; doi:10.1016/j.resplu.2024.100604)
Supplement: Supplementary data 1 [file mmc1.docx]

| **Supplemental Table 1. Equipment** | | | | |
| --- | --- | --- | --- | --- |
| **Accessories** | **Name** | **Company** | **Location** | **Country** |
| Ventilator | Datex | Capnomac Ultima | Helsinki | Finland |
| Left and right ventricular pressure catheter | 5 Fr micro-tip Model SPC 350 | Millar Instruments | TX | USA |
| Carotid arterial ultrasound flowmeter probe | Model 3SB880 | Transsonic Systems Inc. | Ithaca, NY | USA |
| Pulmonary artery catheter | Swan-Ganz CCO | Edwards Lifesciences | Irvine, CA | USA |
| Femoral artery pressure catheter | Fluid filled catheter |  |  |  |
| Femoral vein cooling catheter | 9.3 Fr Cool Line^®^ | Zoll Medical Corporation | CA | USA |
| Laser Doppler flowmetry probe | Model 407 | Perimed AB | Stockholm | Sweden |
| Data acquisition system | PowerLab 16/35 | AD Instruments | Dunedin | New Zealand |
| Software | LabChart 8 | AD Instruments | Dunedin | New Zealand |
| Pulmonary artery monitor | Vigilance II Monitor | Edwards Lifesciences | Irvine, CA | USA |
| Defibrillator | LP12 | Physio Control | Redmond,Wa | USA |
| ICP  Pressure catheter | 7 Fr micro-tip Model SPC 470 | Millar Instruments | TX | USA |
| CO2 sensors | IscAlert | Sensocure AS | Skoppum | Norway |
| Microdialysis catheters | CMA 71-High Cut-Off Brain Microdialysis Catheter | CMA Microdialysis AB | Solna | Sweden |
| Data analysis software | Graphpad Prism 9.3.1 | GraphPad Software | La Jolla, CA | USA |
| Statistics software | IBM SPSS version 26 | SPSS | Chicago, IL | USA |
| **CPB circuit** | **Name** | **Company** | **Location** | **Country** |
| Arterial cannulae | DLP Femoral 14 Fr | Medtronic Inc. | MN | USA |
| Venous cannulae | DLP Jugular 21 Fr | Medtronic Inc. | MN | USA |

**Supplemental Table 2. Average dose during 150-minute observational period after ROSC.**

|  | **Hypothermia** | | | **Normothermia** | | |
| --- | --- | --- | --- | --- | --- | --- |
|  | **Mild hypercapnia**  **(n=10)** | **Normocapnia**  **(n=9)** | **p-value** | **Mild hypercapnia**  **(n=11)** | **Normocapnia**  **(n=9)** | **p-value** |
| **Interventions** |  |  |  |  |  |  |
| Arterial pCO2 (kPa) | 6.9 (6.8, 7.1) | 5.1 (5.0, 5.2) | <0.001 | 6.9 (6.8, 7.1) | 5.2 (5.1, 5.3) | <0.001 |
| Core body temperature(^0^C) | 33.8 (33.3, 34.2) | 33.3 (32.8, 33.8) | <0.001 | 38.7 (38.5, 38.9) | 38.7 (34.4, 38.9) | 1.0 |
|  |  |  |  |  |  |  |
| **Pressure** |  |  |  |  |  |  |
| Mean arterial pressure (mmHg) | 78 (73, 83) | 80 (74, 85) | 0.2 | 82 (77, 82) | 88 (82, 873) | 0.001 |
| Intracranial Pressure (mmHg) | 17 (15, 19) | 15 (12, 18) | 0.004 | 17 (15, 19) | 17 (15, 19) | 0.4 |
| Cerebral Perfusion Pressure (mmHg) | 64 (60, 68) | 62 (56, 69) | 0.3 | 65 (60, 69) | 73 (67, 80) | <0.001 |
| Pressure Reactivity Index (PRx) | 0.08 (0.01, 0.15) | 0.23 (0.16, 0.30) | 0.1 | 0.05 (-0.04, 0.14) | 0.09 (0.04, 0.15) | 0.044 |
|  |  |  |  |  |  |  |
| **Flow** |  |  |  |  |  |  |
| Cardiac Output (litres/min) | 4.8 (4.3, 5.2) | 4.2 (3.9, 4.5) | <0.001 | 5.4 (5.1, 5.8) | 5.2 (4.7, 5.6) | 0.010 |
| Carotid Flow (ml/min) | 265 (244, 286) | 284 (259, 309) | 0.002 | 292 (275, 309) | 260 (235, 285) | <0.001 |
| Cerebral Flow (CF, %) | 79% (63, 95) | 78% (63, 92) | 0.7 | 81% (66, 95) | 84% (72, 95) | 0.2 |
| Brain temperature (^0^C) | 35.5 (35.1, 35.9) | 34.7 (34.1, 35.3) | <0.001 | 39.5 (39.0, 40.0) | 38.1 (36.9, 39.3) | <0.001 |
|  |  |  |  |  |  |  |
| **Metabolism** |  |  |  |  |  |  |
| SvO2 (%) | 76 (73, 79) | 70 (66, 73) | <0.001 | 65 (61, 69) | 65 (60, 70) | 0.8 |
| Brain tissue PtCO_2_ (kPa) | 9.1 (8.6, 9.7) | 7.5 (6.7, 8.3) | <0.001 | 10.9 (10.2, 11.6) | 8.1 (6.8, 9.4) | <0.001 |
| Brain lactate (mM) | 2.3 (2.0, 2.7) | 2.5 (2.1, 3.0) | 0.042 | 1.9 (1.7, 2.2) | 2.1 (1.7, 2.4) | 0.055 |
| Brain pyruvate (µM) | 51 (41, 61) | 72 (50, 93) | <0.001 | 59 (50, 68) | 59 (46, 71) | 0.9 |
| Brain glucose (mM) | 1.2 (0.9, 1.5) | 1.1 (0.9, 1.3) | 0.1 | 1.3 (1.1, 1.4) | 1.1 (1.0, 1.3) | 0.004 |
| Brain glutamate (µM) | 43 (32, 54) | 33 (25, 41) | <0.001 | 20 (14, 26) | 25 (15, 35) | 0.026 |
| Brain glycerol (µM) | 156 (139, 172) | 141 (126, 156) | 0.001 | 149 (129, 169) | 145 (124, 166) | 0.4 |
| Brain Lactate/Pyruvate Ratio | 61 (44, 78) | 48 (34, 62) | 0.004 | 42 (25, 60) | 46 (29, 63) | 0.4 |

Values are given as means with 95% confidence intervals, mean doses are calculated from the Areas Under the Curves.
